# A proliferative probiotic *Bifidobacterium* strain in the gut ameliorates progression of metabolic disorders via microbiota modulation and acetate elevation

**DOI:** 10.1038/srep43522

**Published:** 2017-03-02

**Authors:** Ryo Aoki, Kohei Kamikado, Wataru Suda, Hiroshi Takii, Yumiko Mikami, Natsuki Suganuma, Masahira Hattori, Yasuhiro Koga

**Affiliations:** 1 Institute of Health Sciences, Ezaki Glico Co., Ltd., Nishiyodogawa, Osaka 555-8502, Japan; 2 Graduate School of Frontier Sciences, The University of Tokyo, Chiba 277-8561, Japan; 3 Department of Microbiology and Immunology, Keio University School of Medicine, Tokyo 160-8582, Japan; 4 Graduate School of Advanced Science and Engineering, Waseda University, Tokyo 169-8555, Japan; 5 Department of Infectious Diseases, Tokai University School of Medicine, Isehara, Kanagawa 259-1143, Japan

## Abstract

The gut microbiota is an important contributor to the worldwide prevalence of metabolic syndrome (MS), which includes obesity and diabetes. The anti-MS effects exerted by *Bifidobacterium animalis* ssp. *lactis* GCL2505 (BlaG), a highly proliferative *Bifidobacterium* strain in the gut, and *B. longum* ssp. *longum* JCM1217^T^ (BloJ) were comparatively examined. BlaG treatment reduced visceral fat accumulation and improved glucose tolerance, whereas BloJ had no effect on these parameters. Gut microbial analysis revealed that BlaG exerted stronger effects on the overall bacterial structure of the gut microbiota than BloJ, including enrichment of the genus *Bifidobacterium.* The levels of acetate and glucagon-like peptide-1 were increased by BlaG treatment in both the gut and plasma, but not by BloJ treatment. Correlation analysis suggested that the elevation of gut acetate levels by BlaG treatment plays a pivotal role in the BlaG-induced anti-MS effects. These findings indicated that BlaG, a highly viable and proliferative probiotic, improves metabolic disorders by modulating gut microbiota, which results in the elevation of SCFAs, especially acetate.

Lifestyle changes such as the increased consumption of high-energy foods have greatly contributed to the global prevalence of metabolic syndrome (MS), which includes obesity and diabetes. Recent interest in the gut microbiota has reinforced the concept that our colonic bacteria may not simply reflect lifestyle choices such as diet, but they can also influence energy metabolism. In human and mouse studies, the gut microbiota in obese subjects was characterised by larger populations of Firmicutes and smaller populations of Bacteroidetes as well as a reduction in microbial diversity^1^. Moreover, a high-fat diet (HFD) in mice disrupts intestinal integrity, which exacerbates MS via adipose inflammation^2^,^3^,^4^. Thus, modulation of the gut microbiota is considered an emerging strategy for controlling body weight and insulin sensitivity^4^,^5^,^6^,^7^.

Indigestible carbohydrates derived from the diet are fermented by the gut microbiota and then finally converted to short-chain fatty acids (SCFAs), such as acetate, propionate and butyrate. SCFAs are absorbed via the colonic epithelium, and acetate—the most abundant SCFA—achieves concentrations of 19–160 μM in peripheral blood, whereas propionate and butyrate reach 1–13 μM and 1–12 μM^8^, respectively. Recently, it was revealed that SCFAs have distinct actions relevant to energy homoeostasis in addition to serving as host energy sources. Acetate activates GPR43, a G-protein-coupled receptor (GPCR), on adipocytes and suppresses insulin signalling, which inhibits fat accumulation in adipocytes and promotes the metabolism of unincorporated lipids and glucose in other tissues^9^. In addition, it reduces appetite through a central hypothalamic mechanism^10^, and pancreatic GPR43 signalling potentiates β-cell function^11^. Furthermore, Tolhurst *et al*.^12^ revealed that GPR43 on intestinal L-cells is stimulated by SCFAs, and induces the secretion of glucagon-like peptide-1 (GLP-1), a gut-derived peptide known to modulate satiety and glucose homoeostasis^13^,^14^.

Probiotics are defined as ‘live microorganisms that, when administered in adequate amounts, confer a health benefit on the host’^15^, and *Bifidobacterium* and *Lactobacillus* spp. are representative examples of such probiotic microorganisms. Several probiotics and their mixture have been reported to improve MS^16^,^17^,^18^,^19^,^20^. In some of these reports, several probiotics were found to improve MS by modulating the composition of the gut microbiota or its metabolites^16^,^18^. However, very few studies have comprehensively investigated the effects of probiotics on the composition of the gut microbiota, its metabolites, and host metabolic parameters. Furthermore, the properties of probiotics involved in these effects remain unclear.

Thus, the present study investigated the effects of probiotic *Bifidobacterium* treatment on MS and on the underlying mechanisms in order to elucidate the properties of probiotics involved in the anti-MS effects. To achieve this, we used two *Bifidobacterium* strains, *B. animalis* ssp. *lactis* GCL2505 (BlaG) and *B. longum* ssp. *longum* JCM1217^T^ (BloJ), which have different properties in the gut, and intensively investigated their effects on metabolic parameters, global changes in the gut microbiota, and SCFA levels in mice. BlaG was originally isolated from the faeces of a healthy adult, and is reported to be a probiotic strain capable of proliferating in the gut^21^,^22^. BloJ is a type strain of *Bifidobacterium longum* that has been widely used in commercial probiotics^23^ and is reported to protect the host from enteropathogenic infection through the production of acetate^24^, but is not capable of proliferating in the gut^22^.

## Results

### *B. lactis* GCL2505 treatment improved glucose tolerance

**Figure 1:**
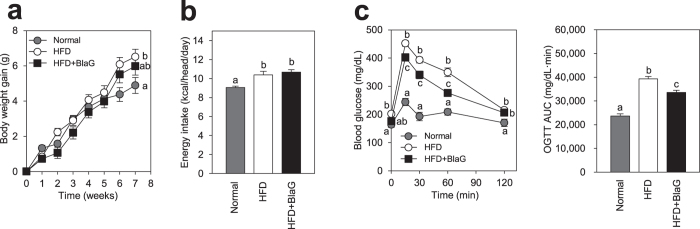
*Bifidobacterium animalis* ssp. *lactis* GCL2505 (BlaG) treatment improved glucose tolerance in mice fed a high-fat diet (Experiment 1). (**a**) Body weight gain. (**b**) Daily energy intake. (**c**) Blood glucose levels and the area under the curve after oral glucose challenge (2 g/kg body weight) at 6 weeks of the BlaG treatment. Data represent the mean ± SEM. Statistical analyses were performed using the Tukey–Kramer multiple comparison test. Different letters next to bars indicate a significant difference (P < 0.05).

In Experiment 1, a high-fat diet (HFD) significantly increased body weight (Fig. 1a), energy intake (Fig. 1b), blood glucose (Fig. 1c), and plasma insulin levels after glucose challenge (Supplementary Fig. S2). Glucose tolerance was significantly improved by BlaG treatment in HFD-fed mice (Fig. 1c) and *ob/ob* mice (Experiment 3, Supplementary Fig. S1). BlaG treatment did not affect weight gain, energy intake, plasma insulin levels, or intestinal integrity-related parameters including the permeability of fluorescein-conjugated dextran or the gene expression of tight junction proteins (Fig. 1 and Supplementary Fig. S2).

### *Bifidobacterium* strain-specific effects on metabolic parameters

**Figure 2:**
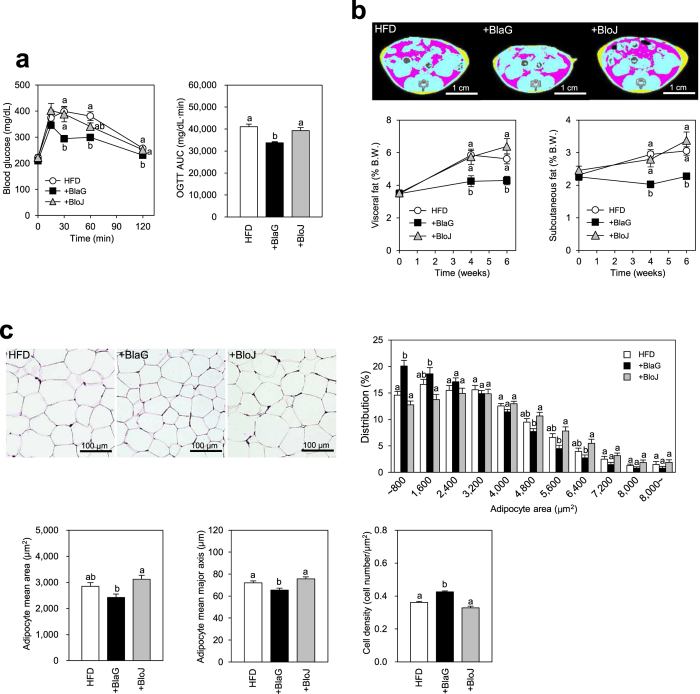
Comparison of the BlaG treatment and *Bifidobacterium longum* JCM1217^T^ (BloJ) treatment to improve glucose tolerance and body fat accumulation in HFD mice (Experiment 2). (**a**) Blood glucose levels and the area under the curve after oral glucose challenge (2 g/kg body weight) after the bifidobacteria treatment for 6 weeks. (**b**) Representative computed tomography (CT) images after 6 weeks of probiotic treatments and serial assessment of CT-estimated proportions of visceral and subcutaneous fat weight to body weight after 0–6 weeks of probiotic treatment. The pink and yellow areas in CT images represent the visceral and subcutaneous fat, respectively. (**c**) Representative epididymal fat tissue staining, cell area distribution, mean area, mean major axis, and cell density of epididymal adipocytes after 7 weeks of bifidobacteria treatments. Data represent the mean ± SEM. Statistical analyses were performed using the Tukey–Kramer multiple comparison test. Different letters next to bars indicate a significant difference (P < 0.05).

To investigate the properties of BlaG that improve glucose tolerance in the host, we compared the effect of BlaG with that of BloJ on several metabolic parameters of mice in Experiment 2. BlaG treatment significantly improved glucose tolerance compared with HFD-fed controls; however, BloJ treatment had no such effect (Fig. 2a). X-ray computed tomography (CT) analysis revealed that the accumulations of both visceral and subcutaneous fat were significantly decreased by BlaG treatment at 4 and 6 weeks after the start of treatment. However, BloJ treatment had no effect on the accumulation of either type of body fat (Fig. 2b). Histological analysis of epididymal adipocytes showed a greater proportion of smaller adipocytes in the BlaG-treated group than in the HFD-fed control group (Fig. 2c). On the other hand, no difference in adipocyte size was observed between the BloJ-treated and HFD-fed control groups. These results indicated that BlaG but not BloJ had effective anti-MS effects when administered to HFD-fed mice. Neither the plasma insulin levels after glucose challenge nor the triglyceride levels were affected by BlaG or BloJ treatment in HFD-fed mice (Supplementary Fig. S3).

### *B. lactis* GCL2505 treatment altered the overall structure of gut microbiota

**Figure 3:**
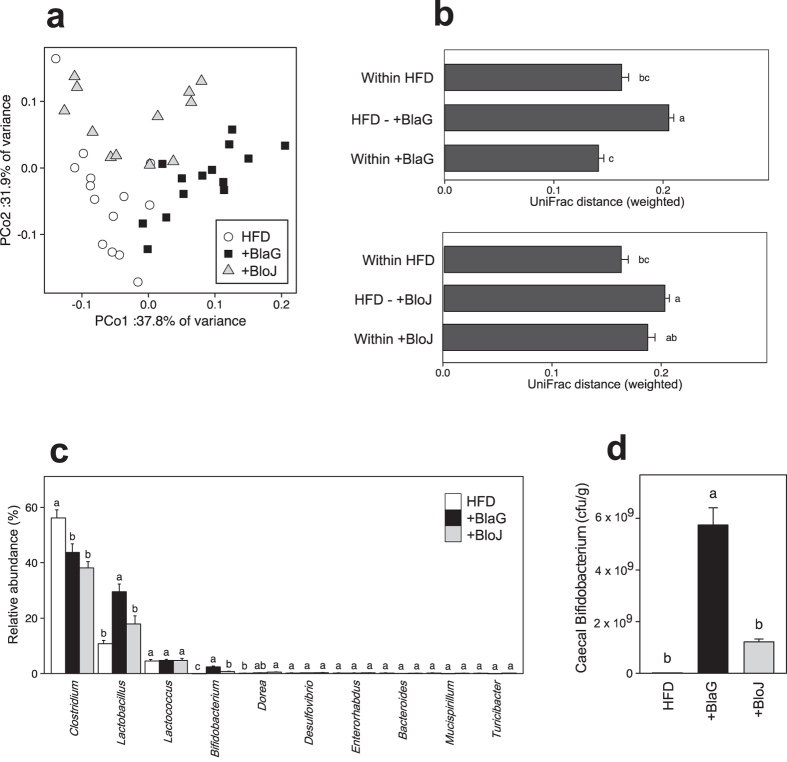
Analysis of the microbiota in the contents of the caecum. (**a**) Principal coordinate analysis plot generated using a weighted UniFrac metric. The two components explained 69.7% of the variance. (**b**) Weighted UniFrac distance metric in the HFD-fed control, the BlaG-treated, and the BloJ-treated groups. (**c**) Bacterial composition at the genus level in the samples. For genus-level assignment of 16 S reads (16 S rRNA gene V1–V2 region), a 94% sequence identity threshold was applied. The vertical axis represents the relative abundance (%) of each genus in the microbiota. (**d**) The number of *Bifidobacterium* in caecal samples quantified by quantitative polymerase chain reaction. Data represent the mean ± SEM. Tukey–Kramer multiple comparison test was used. Different letters next to bars indicate a significant difference (P < 0.05).

We compared the overall bacterial community structure of the gut microbiota by using 16 S rRNA gene pyrosequencing in Experiment 2. A principal coordinate analysis based on the weighted UniFrac metric revealed that the BlaG-treated group formed a distinct cluster clearly set apart from the HFD-fed control group (Fig. 3a). In addition, the mean distance in the mixture of HFD-fed control and the BlaG-treated groups was significantly higher than in the HFD or BlaG groups (Fig. 3b). However, the distance in the mixture of HFD-fed control and BloJ-treated groups was no different to that of the BloJ groups. Moreover, cluster analysis against the weighted UniFrac metric also revealed a clear separation of the HFD-fed control group from the BlaG-treated group in accordance with the bar chart (Supplementary Fig. 4b). These results indicated that BlaG treatment exerted a significantly greater influence on the overall bacterial community structure of gut microbiota compared with BloJ treatment in HFD-fed mice.

### Composition of gut microbiota

In bacterial composition analysis at the genus level, the abundance of both *Lactobacillus* and *Bifidobacterium* was significantly greater in the BlaG-treated group than in the HFD-fed controls or the BloJ-treated group (Fig. 3c). Quantitative polymerase chain reaction (qPCR) also demonstrated that the number of *Bifidobacterium* cells was significantly greater in the BlaG-treated group than in the BloJ-treated group (Fig. 3d). Species-level analysis revealed that both *L. johnsonii* and *B. animalis* were more abundant in the BlaG-treated group than in the control group (Supplementary Fig. S4e). The number of administered *Bifidobacterium* strains in the gut was quantified by qPCR using species-specific primers, namely, *B. lactis*-specific primers for the BlaG-treated group and *B. longum*-specific primers for the BloJ group. In the BlaG-treated group, the number of *B. lactis* was 2.6 ± 0.3 × 10^9^ cfu/g, whereas the number of *B. longum* in the BloJ-treated group was far less at 0.46 ± 0.17 × 10^9^ cfu/g.

### *B. lactis* GCL2505 treatment elevated both acetate and GLP-1 levels, which are associated with metabolism parameters

**Figure 4:**
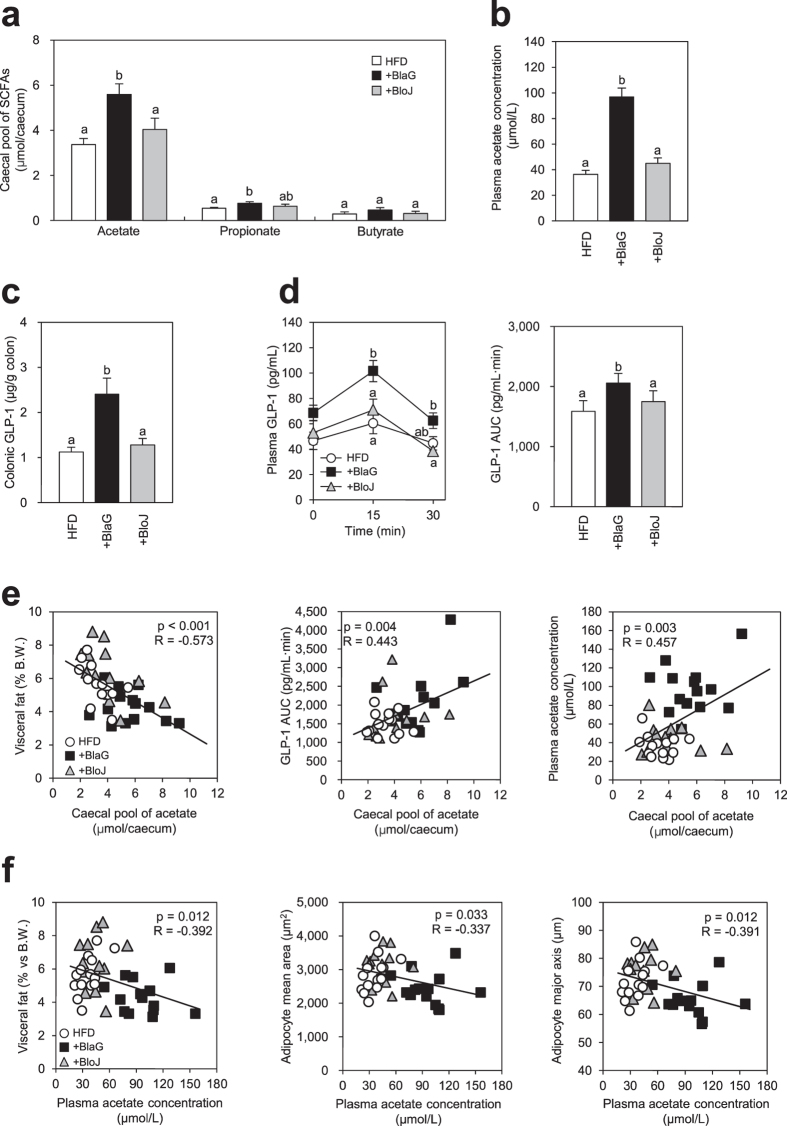
Effect of bifidobacteria treatment on short-chain fatty acid (SCFA) levels, glucagon-like peptide-1 (GLP-1) secretion, and the correlation between the two variables. (**a**) SCFA levels in the caecum after 7 weeks of probiotic treatments. (**b**) Plasma acetate concentration. (**c**) Colonic GLP-1 levels measured using enzyme-linked immunosorbent assay. (**d**) Plasma GLP-1 levels and the area under the curve 0–30 min after oral glucose challenge in mice fed a high-fat diet with 6 weeks of probiotic treatments. (**e**) Correlation analysis between caecal acetate levels and visceral fat accumulation, plasma acetate levels and GLP-1 levels. (**f**) Correlation analysis between plasma acetate levels and visceral fat accumulation and adipocyte size. Pearson’s *R* correlation and corresponding P value are presented. Data represent the mean ± SEM. Tukey–Kramer multiple comparison test was used to assess the differences between the groups. Different letters next to bars indicate a significant difference (P < 0.05).

We measured the amount of SCFAs in the gut. The caecal pools of acetate and propionate were significantly higher in the BlaG-treated group than in the HFD-fed control group (Fig. 4a). Similar elevation of caecal acetate by BlaG treatment was also observed in the preliminary *ob/ob* mice model (Experiment 3, Supplementary Fig. S1e). However, these SCFAs were not markedly elevated by BloJ treatment in the HFD-fed mice. The plasma acetate concentration was also significantly higher in the BlaG-treated group than in the HFD-fed control or BloJ-treated groups (Fig. 4b). Compared with the HFD-fed control group, a significant elevation of colonic GLP-1 secretion was observed in the BlaG-treated group, but not in the BloJ-treated group (Fig. 4c). In addition, plasma GLP-1 levels after glucose challenge were significantly elevated only by BlaG treatment (Fig. 4d). We performed correlation analyses to investigate whether the caecal SCFAs affect metabolic parameters and/or GLP-1 secretion. The level of caecal acetate correlated negatively with the amount of visceral fat but positively with plasma GLP-1 levels (Fig. 4e). Moreover, the concentration of plasma acetate correlated positively with the caecal pool of acetate, but negatively with both the amount of visceral fat and adipocyte size (Fig. 4e,f).

### Relationship between gut microbiota composition and SCFA/MS-related parameters

**Figure 5:**
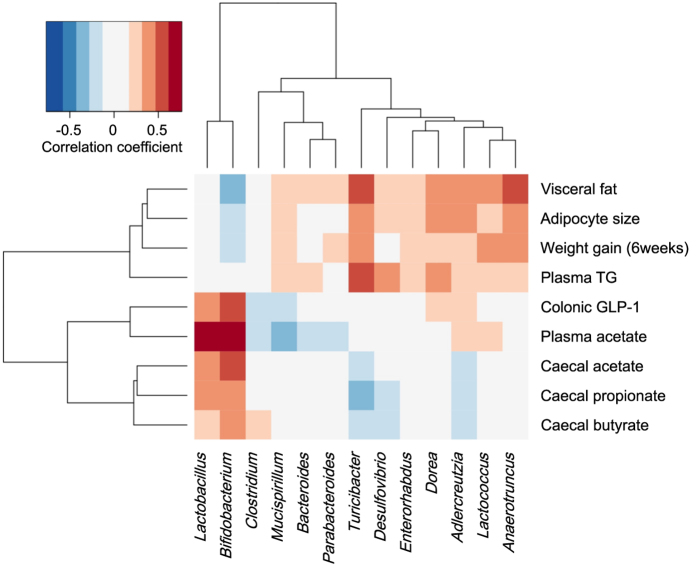
Correlation between the relative abundance of genera and metabolic parameters in mice. Pearson’s correlation coefficients are represented by colour ranging from blue (negative correlation, −1) to red (positive correlation, 1). Data on genus reads used for the analysis represented 73.7% of the total reads of microbiota.

We next assessed the correlation between the relative abundance of dominant bacterial genera and SCFAs/ MS-related parameters to identify the genera that might contribute to the production of SCFA or the anti-MS effects (Fig. 5). At the genus level, the relative abundance of *Bifidobacterium* correlated positively with caecal acetate, plasma acetate and colonic GLP-1 levels (r = 0.54, 0.76 and 0.60, respectively, all P < 0.001), and negatively with the amount of visceral fat (r = −0.31, P < 0.05). The abundance of *Lactobacillus* also correlated with caecal acetate, plasma acetate and colonic GLP-1 levels (r = 0.67, 0.42 and 0.43, respectively, all P < 0.01). The other genera showed no correlation with SCFA levels. At the species level, correlation and network analyses revealed that the abundance of *B. animalis, L. johnsonii* and *L. reuteri* correlated positively with caecal SCFA, plasma acetate level and GLP-1 (Supplementary Figs S5 and 6).

## Discussion

Although numerous reports indicate that several probiotics improve MS^16^,^17^,^18^,^19^,^20^, the properties of probiotics that are involved in these anti-MS effects remain unclear. In the present study, BlaG treatment improved glucose tolerance in HFD-fed mice as well as in *ob/ob* mice (Fig. 1 and Supplementary Fig. S1). We conducted a comparative analysis using both BlaG and BloJ to investigate the mechanism(s) underlying the anti-MS effect by BlaG. The results showed that only BlaG improved glucose metabolism, concomitantly suppressing the accumulation of body fat and adipocyte hypertrophy (Fig. 2).

Several studies have reported that the anti-obesity effect of probiotics is related to changes in the gut microbiota^16^,^18^,^25^. We thus hypothesised that BlaG exerts an influence on the gut microbiota that differs from that exerted by BloJ because it has a greater proliferative activity in the gut than BloJ^22^. Interestingly, 16 S rRNA pyrosequencing analysis of caecal contents revealed that BlaG exerted a significantly greater influence on the overall bacterial structure of gut microbiota than BloJ (Fig. 3b and Supplementary Fig. S4b). Analysis of the bacterial composition at genus-level revealed that the relative abundances of the genera *Bifidobacterium* and *Lactobacillus* were remarkably enriched in the gut microbiota of the BlaG-treated mice (Fig. 3c). Indeed, qPCR analysis demonstrated that the number of *B. lactis* in the BlaG-treated group was more than five-fold greater than that of *B. longum* in the BloJ-treated group following treatment with the same cell numbers of each probiotic strain (see results). Consequently, BlaG treatment resulted in approximately a four-fold increase in the number of *Bifidobacterium* compared with BloJ treatment (Fig. 3d). These results demonstrated that BlaG proliferated effectively in the gut in HFD-fed mice because there were a greater number of *Bifidobacterium* cells present in BlaG-treated group, which agrees with previous reports^21^,^22^. Therefore, the highly proliferative probiotic strain BlaG altered the overall bacterial structure of the gut microbiota, probably through efficient proliferation, resulting in the improvement of MS, whereas the low numbers of *Bifidobacterium* strain BloJ had little influence on the microbiota or host metabolism.

The dominant metabolites of *Bifidobacterium* and *Lactobacillus* are acetate/lactate and lactate, respectively. Lactate is eventually metabolised to other organic acids including SCFAs by gut microbes^26^. Hence, we next focused on the changes in levels of SCFAs in the gut and plasma following probiotic treatment. Caecal and plasma acetate levels were significantly elevated by BlaG treatment, whereas the levels were unchanged by BloJ treatment (Fig. 4a,b). Similar elevation of caecal acetate by BlaG treatment was also observed in the *ob/ob* mice model (Supplementary Fig. S1e). Correlation analysis between the abundances of the gut microbiome and SCFAs revealed that genus *Bifidobacterium* and genus *Lactobacillus* correlated positively with SCFAs levels (Fig. 5). These data suggested that the enrichment of *Bifidobacterium* and *Lactobacillus* in the BlaG-treated mice might play a major role in accelerating the production of SCFAs in the gut. Because *Lactobacillus* mostly produces lactate as an end product of carbohydrate metabolism, the increase in *Bifidobacterium* appeared to have mainly contributed to the elevation of acetate in the BlaG-treated mice.

It has been recently demonstrated that SCFAs regulate energy homoeostasis via GPCRs^9^,^27^. GPR41 is activated equally by propionate and butyrate, whereas GPR43 is more responsive to acetate and propionate than to butyrate^28^,^29^. In the intestinal epithelium, SCFAs were found to stimulate GPR43, which resulted in the secretion of GLP-1, a gut-derived hormone that plays a significant role in energy metabolism^9^. In the present study, BlaG treatment significantly enhanced GLP-1 levels in both the colon and plasma, whereas no such effect was observed in the BloJ-treated mice (Fig. 4c,d). In addition, colonic GLP-1 levels correlated positively with luminal acetate levels (Fig. 4e) but not with propionate or butyrate levels (data not shown). These results implied that enhanced luminal acetate levels following BlaG treatment stimulated GLP-1 secretion in the colon, possibly via GPR43.

Kimura *et al*.^9^ reported that GPR43 signalling in adipocytes inhibits insulin signalling, and results in the suppression of fat accumulation in adipose tissue and an improvement in systemic insulin resistance. In the present study, BlaG treatment resulted in the elevation of both plasma and caecal acetate levels (Fig. 4a,b) in conjunction with improved glucose tolerance and less body fat accumulation. On the other hand, acetate levels showed a significant negative correlation with the amount of visceral fat and adipocyte size (Fig. 4e,f). These results suggested that the elevation in plasma acetate following BlaG treatment was associated with a reduction in the size of adipocytes, probably through enhanced insulin sensitivity via GPR43 signalling, which resulted in improved glucose tolerance and less fat accumulation. Together with the elevation in GLP-1 expression following BlaG treatment, our findings suggest that the enhanced production of gut acetate probably played a pivotal role in the anti-MS effects observed.

It has been reported that GLP-1 and GPR43 signalling potentiates insulin secretion by beta cells in the pancreas^13^. In addition, GLP-1 and acetate are reported to control appetite via the central nervous system^10^,^30^. However, bifidobacteria treatment did not affect either insulin secretion or food intake in this study (Fig. 1 and Supplementary Figs S2 and 3). Further research is therefore required to investigate why the elevation of both GLP-1 secretion and plasma acetate in this study did not lead to increased insulin secretion or appetite suppression.

Several possible relationships between metabolic parameters and intestine-related parameters, such as intestinal integrity, and between the diversity of the gut microbiota and the Bacteroidetes/Firmicutes ratio have been reported^3^,^31^. Some reports have indicated that increases in the number of bifidobacteria following probiotic or prebiotic treatment might restore impaired intestinal integrity^4^,^5^. In the present study, there were no changes in intestinal integrity-related parameters (Supplementary Fig. S2c and d) or microbial diversity (Supplementary Fig. S4a). Moreover, phylum-level analysis of the gut microbiota in this study demonstrated that BlaG treatment significantly elevated the abundance of phylum Actinobacteria but did not affect the Bacteroidetes/Firmicutes ratio (Supplementary Fig. S4d). It appears unlikely that intestinal integrity, microbial diversity, or the Bacteroidetes/Firmicutes ratio is involved in the anti-MS effects mediated by BlaG treatment.

In conclusion, *B. lactis* GCL2505, a highly viable and proliferative probiotic, exerted anti-MS effects, such as improved glucose tolerance and the suppression of visceral fat accumulation, via changes in the overall bacterial structure of the gut microbiota and elevations in the levels of SCFAs, especially acetate.

## Methods

### Preparation of *Bifidobacterium* strains

BlaG was obtained from Ezaki Glico Co., Ltd. and cultured in Gifu anaerobic medium broth (Nissui) supplemented with up to 1% glucose. BloJ was obtained from the Japan Collection of Microorganisms (RIKEN BioResource Center) and cultured in MRS broth (Merck). To prepare the probiotic treatments, these strains were cultured daily under anaerobic conditions using the AnaeroPack Kenki (Mitsubishi Gas Chemical Co.). Cultures were washed and suspended in phosphate-buffered saline (PBS) or saline for the duration of the treatments.

### Ethics of animal treatments

All experimental procedures were approved by the Tokai University Animal Experimentation Committee and the Institutional Animal Care and Use Committee of Ezaki Glico Co., and were performed in accordance with either the Guidelines for the Care and Use of Animals for Scientific Purposes at Tokai University or the Guidelines for Proper Conduct of Animal Experiments (Science Council of Japan).

### Experiment 1

Five-week-old male C57BL/6 J mice (SLC Inc.) were housed in a controlled environment (12-h/12-h light-dark cycle). Mice were fed a normal diet (10% of calories from fat; D12450B, Research Diet Inc.) (Normal group, n = 12) or a HFD (45% of calories from fat; D12451, Research Diets Inc.). After 2 weeks, the HFD-fed mice were divided into HFD-fed control (n = 12) and BlaG-treated (n = 12) groups based on their body weights. Mice in the BlaG-treated groups received oral administration of the appropriate BlaG strain (1 × 10^9^ colony forming units/day) daily for 7 weeks. Mice in the normal or HFD-fed control groups were given PBS daily. OGTT was performed 6 weeks after BlaG treatment (see below). After a further week, intestinal permeability testing was performed (see Supplementary methods) and all mice were sacrificed as described below. The experimental design is shown in Supplementary Fig. S7a.

### Experiment 2

Five-week-old male C57BL/6 J mice (SLC Inc.) were housed in a controlled environment (12-h/12-h light-dark cycle). All mice were fed a HFD (D12451). After 2 weeks, the mice were divided into HFD-fed control (n = 12–14), BlaG (n = 12–14) and BloJ (n = 11–12) -treated groups based on their body weights and fat composition measured by X-ray CT (see below). Mice in the BlaG- or BloJ-treated groups received oral administration of the appropriate *Bifidobacterium* strain (1 × 10^9^ colony forming units/day) daily for 7 weeks. Mice in the HFD-fed control group were given saline daily. At 0, 4, and 6 weeks after probiotics treatment started, body fat composition was analysed using X-ray CT (see below). OGTTs were performed 6 weeks after probiotics treatment commenced (see below). All mice were sacrificed at the end of the trial as described below. This experiment was performed in duplicate and the experimental design is shown in Supplementary Fig. S7b.

### Oral glucose tolerance tests

Oral glucose tolerance tests were performed as follows. Mice were fasted for 6 h and then injected with glucose by gavage (2 g/kg glucose). Blood glucose content was determined with a glucose meter (Glucose Pilot, Aventir Biotech) using blood collected from the tip of the tail vein. In addition, plasma samples were collected after the glucose challenge to assess glucose-stimulated GLP-1 and insulin expression.

### Body fat composition analysis

Body fat composition was analysed by the method described by Lubura *et al*.^32^ with some modifications. In brief, mice were anaesthetised by inhalation of isoflurane and scanned using a LaTheta (LCT-100) experimental animal X-ray CT system (Aloka). Contiguous 1-mm slice images between the proximal end of lumbar vertebra L1 and the distal end of L6 were used for quantitative assessment using LaTheta software (version 2.10, Aloka). Subcutaneous and visceral fat levels were evaluated quantitatively.

### Sample collection

Mice were killed in a CO_2_ atmosphere after a 4-h fast, and blood samples and tissues were harvested for further analysis. Blood samples were collected into tubes containing heparin (Ajinomoto Co.) following cardiac puncture, and plasma was stored at −80°C after centrifugation at 3,000 × *g* for 15 min at 4 °C. Caecal contents and the colon segments were precisely dissected and stored at −80°C for further analysis. Colon segments were stored in RNAlater (Sigma-Aldrich) at −80°C until mRNA expression analysis. The epididymal fat was stored in 10% neutral formalin for histological analysis.

### Biochemical analysis

For plasma GLP-1 quantification, blood samples were collected in test tubes containing a dipeptidyl peptidase IV inhibitor (Millipore). Colonic GLP-1 was extracted according to the method described by Cani *et al*.^33^. The homogenate was centrifuged at 2000 × *g* for 20 min at 4 °C, and the supernatant fraction was decanted and diluted 1000-fold in assay buffer. The active GLP-1 concentration was determined using an enzyme-linked immunosorbent assay (ELISA) method (Active GLP-1 ELISA Kit, Shibayagi). Plasma and caecal acetate levels were enzymatically measured in duplicate using a commercial kit (Acetate Colorimetric Assay Kit, Sigma-Aldrich). The analysis of other caecal SCFAs was performed as follows: Caecal samples were suspended in 5% (w/v) metaphosphate-PBS (49 mL/g sample). Suspensions were subsequently filtered using a 3-kDa MWCO spin column (Pall Corporation) and analysed on a gas chromatography-flame ionisation detector system (Shimadzu GC2014, Shimadzu, Kyoto, Japan) equipped with a glass column packed with 60/80 SHINCARBON A coated with Themon-3000 (Shinwa-kakou). Plasma triglyceride (Wako) and insulin (Ultrasensitive Mouse Insulin ELISA, Mercodia) levels were determined using the corresponding commercial kits.

### Adipose tissue histology

Epididymal fat was fixed in 10% neutral formalin, processed in paraffin blocks, sectioned to a thickness of 4 μm, and stained with haematoxylin and eosin. Slides were scanned using a BIOREVO BZ-9000-Generation-II microscope (KEYENCE Co.), and the sizes of 700–1500 adipocytes were measured using BZ-II-Analyzer software (version 2.2, KEYENCE Co.). The number of adipocytes per total area of counted adipocytes was determined as the cell density.

### 16 S rRNA metagenomic analysis

The extraction of bacterial DNA from the samples of caecal contents was performed as described previously^34^. The hypervariable V1–V2 region of the 16 S gene was amplified by PCR with barcoded 27Fmod (5′-AGRGTTTGATYMTGGCTCAG-3′) and reverse primer 338R^35^ (5′-TGCTGCCTCCCGTAGGAGT-3′) and then sequenced using the 454 GS FLX Titanium or 454 GS Junior system (Roche Applied Science). Operational taxonomic unit (OTU) clustering, taxonomy assignment and UniFrac analysis were performed as described previously^36^.

### Quantitative PCR analysis of the microbiota in the caecum

Bacterial DNA was extracted from the contents in the caecum as described previously^21^. The bacterial strains used as standard curves as well as the primers and amplification programs are listed in Supplementary Table 1. Quantitation of specific bacterial counts, the bacterial strains used as standard curves, primers and amplification programs are also listed in Supplementary Table 1.

### Statistical analysis

Results are presented as means ± SEM. Statistical evaluation of multiple groups was performed using one-way ANOVA with posthoc Tukey Kramer multiple comparison testing. Statistical evaluation of two groups was performed using the Student t-test. Correlation analyses were conducted using Pearson linear regression. A value of P < 0.05 was considered statistically significant. All statistical analyses were conducted using the open-source software program R, Version 3.1.1 (http://cran.r-project/org).

## Additional Information

**How to cite this article:** Aoki, R. *et al*. A proliferative probiotic *Bifidobacterium* strain in the gut ameliorates progression of metabolic disorders via microbiota modulation and acetate elevation. *Sci. Rep.* **7**, 43522; doi: 10.1038/srep43522 (2017).

**Publisher's note:** Springer Nature remains neutral with regard to jurisdictional claims in published maps and institutional affiliations.

## Supplementary Material

Supplementary Information
