# Supplementary material for: A proliferative probiotic Bifidobacterium strain in the gut ameliorates progression of metabolic disorders via microbiota modulation and acetate elevation
Source: Sci Rep. 2017 Mar 2;7:43522. doi: 10.1038/srep43522 (PMC5333160; doi:10.1038/srep43522)
Supplement: Supplementary Information [file srep43522-s1.doc]

**A proliferative probiotic *Bifidobacterium* strain in the gut ameliorates progression of metabolic disorders via microbiota modulation and acetate elevation**

Ryo Aoki^1,2,+*^, Kohei Kamikado^1,+^,Wataru Suda^2,3^, Hiroshi Takii^1^, Yumiko Mikami^1^, Natsuki Suganuma^1^, Masahira Hattori^2,4^ and Yasuhiro Koga^5^

^1^Institute of Health Sciences, Ezaki Glico Co., Ltd., Nishiyodogawa, Osaka 555-8502, Japan

^2^Graduate School of Frontier Sciences, The University of Tokyo, Chiba 277-8561, Japan

^3^Department of Microbiology and Immunology, Keio University School of Medicine, Tokyo 160-8582, Japan

^4^Graduate School of Advanced Science and Engineering, Waseda University, Tokyo 169-8555, Japan

^5^Department of Infectious Diseases, Tokai University School of Medicine, Isehara, Kanagawa 259-1143, Japan

*Corresponding author:

Ryo Aoki

Email address: aoki-ryo@glico.co.jp

+these authors contributed equally to this work

**Supplementary Figure S1.** **Effect of *Bifidobacterium animalis* ssp. *lactis* GCL2505 (BlaG) in *ob/ob* mice model (Experiment 3)**. (a) Body weight gain during 7 weeks of BlaG treatment. (b) Six-hour fasting plasma triglyceride levels. (c) Blood glucose levels and the area under the curve after oral glucose challenge (2 g/kg body weight) following BlaG treatment for 6 weeks. (d) The number of specific bacteria in caecal samples obtained by quantitative polymerase chain reaction. (e) Levels of SCFAs in the caecum after 7 weeks of BlaG treatment. Data represent the means ± SEM. Statistical analyses were performed using Student t test. Different letters next to bars indicate a significant difference (P < 0.05).

**
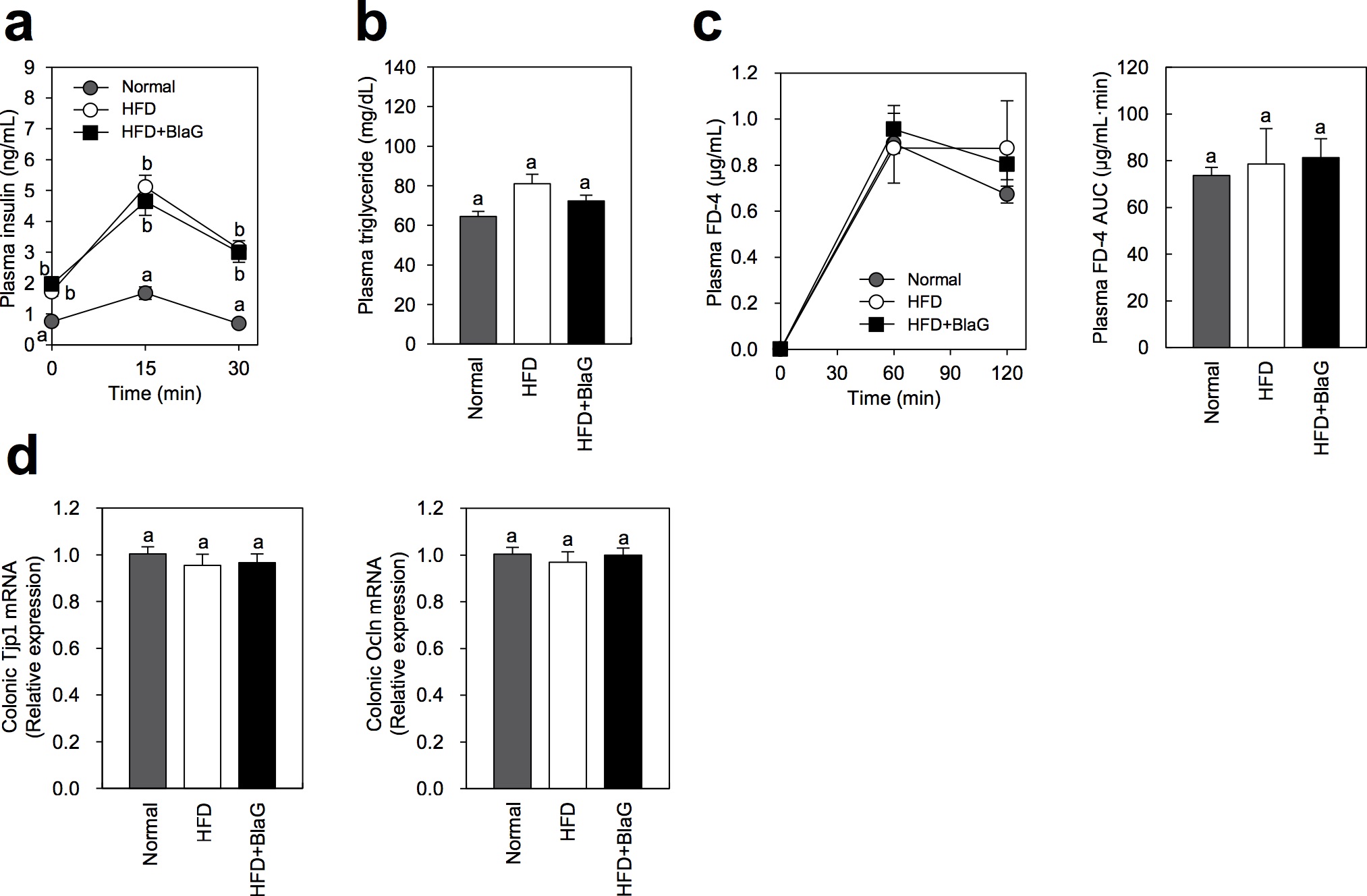
**

**Supplementary Figure S2. BLaG treatment did not affect intestinal permeability or insulin secretion in high-fat diet (HFD) fed mice (Experiment 1).** (a) Plasma insulin levels and the area under the curve (AUC) 0–30 min after oral glucose challenge (2 g/kg body weight). (b) Plasma fluorescein isothiocyanate-dextran (molecular weight = 4 kDa: FD-4, Sigma-Aldrich) levels and the AUC after oral FD-4 treatment following BlaG treatment for 7 weeks. (c) Colonic mRNA expression of intestinal integrity-related gene (*Tjp1*; Tight junction protein-1, *Ocln*; Occludin) was measured using primers and programs described in Supplementary Table 2, and data are presented as the ratio of expression to that of the control group normalised by *Hprt1* mRNA expression. Data represent the means ± SEM. Statistical analyses were performed using the Tukey–Kramer multiple comparison test. Different letters indicate significant difference (P < 0.05).

**
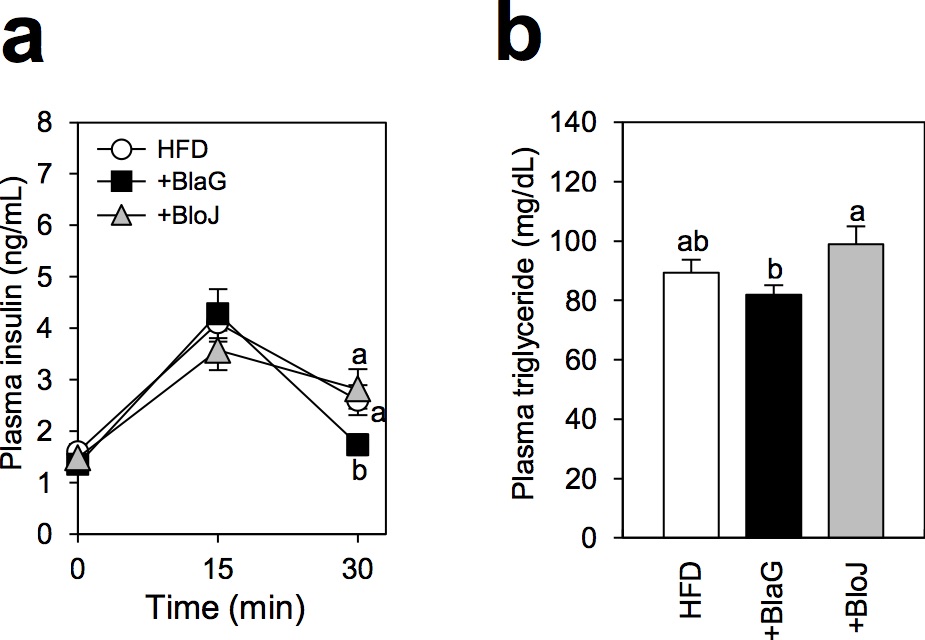
**

**Supplementary Figure S3. The effects of BlaG or *Bifidobacterium longum* JCM1217^T^ (BloJ) treatment on plasma insulin and triglyceride levels in HFD-fed mice (Experiment 2).** (a) Plasma insulin levels and the area under the curve 0–30 min after oral glucose challenge (2 g/kg body weight). (b) Plasma triglyceride levels. Data represent the means ± SEM. Statistical analyses were performed using the Tukey–Kramer multiple comparison test. Different letters indicate significant difference (P < 0.05).

**
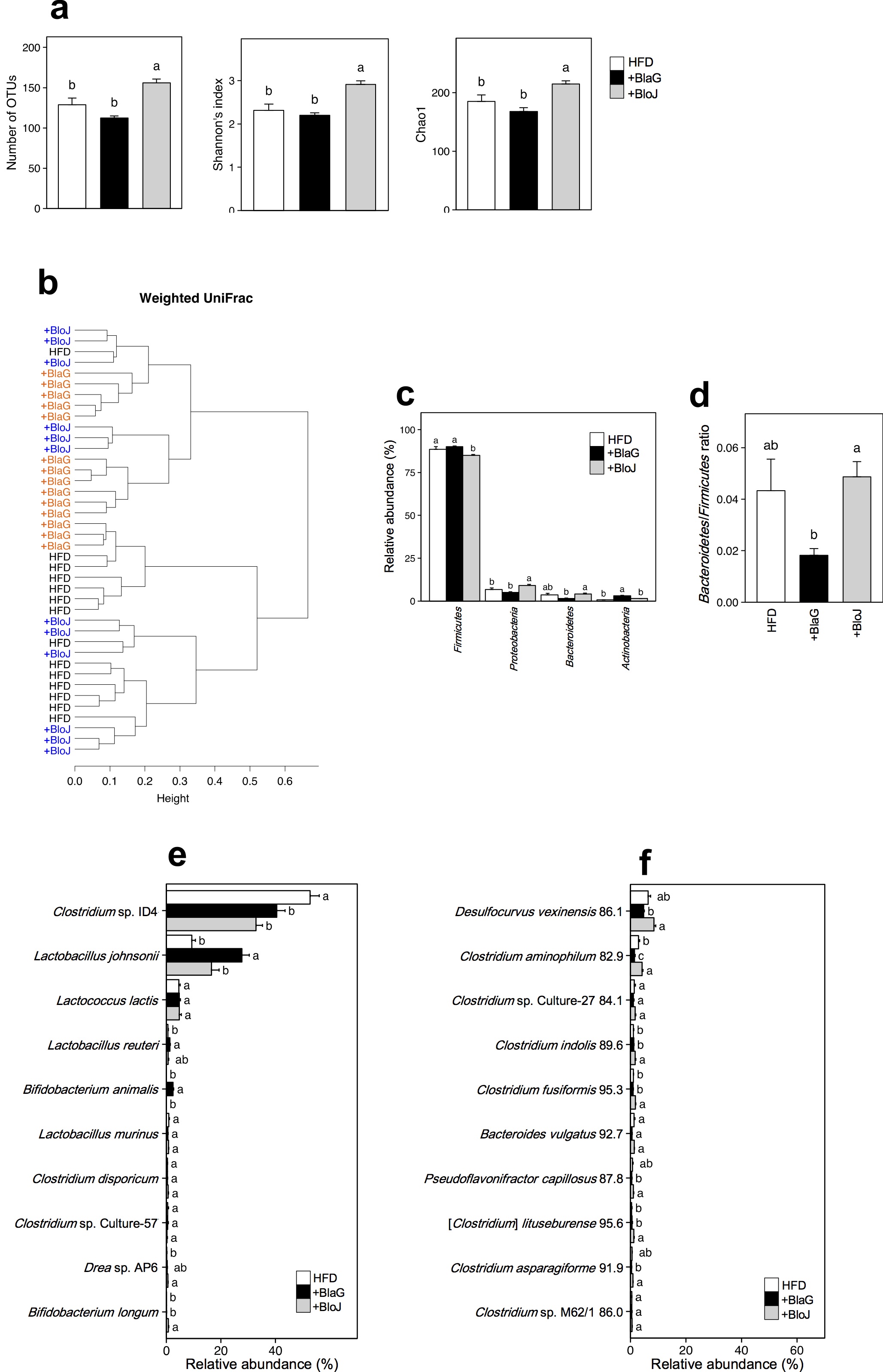
**

**Supplementary Figure S4. Microbial structures of caecal contents in HFD-fed mice.** Operational taxonomic unit (OTU)/species diversity and richness in the gut microbiota. (a) OTU-based microbial richness and diversity. OTUs generated by clustering 3,000 16S reads of gut microbiota, estimated OTU numbers obtained from Chao1 extrapolation of the observed OTU numbers, and the Shannon index calculated from the observed OTU numbers. (b) Cluster dendrogram generated using the weighted UniFrac metric. (c) Bacterial composition at the phylum level in gut microbiota samples. For phylum level assignment, a 70% sequence identity threshold was applied. The vertical axis represents the relative abundance (%) of each genus in the microbiota (d) Bacteroidetes/Firmicutes ratio calculated using the phylum abundance. (e) Bacterial composition at the species level in gut microbiota samples. For species level assignment, a 96% sequence identity threshold was applied. (f) Bacterial composition at the undefined OTUs in gut microbiota samples. Undefined OTUs were expressed as the best-hit species with identity. Data represent the means ± SEM. Tukey–Kramer multiple comparison test was used. Different letters indicate significant difference (P < 0.05).

**
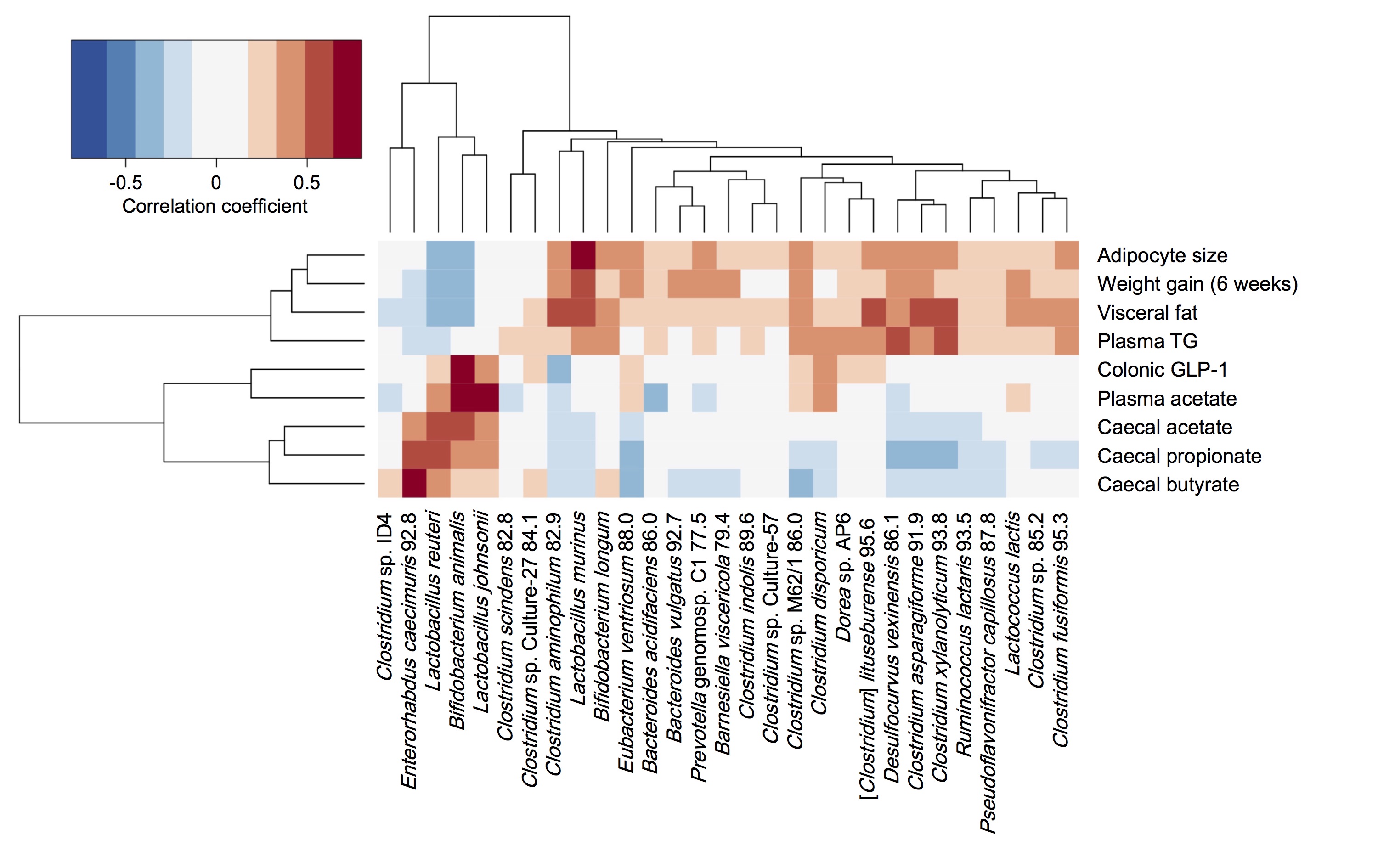
**

**Supplementary Figure S5. Correlation between the relative abundance of species and metabolic parameters in HFD-fed mice.** Pearson’s correlation coefficients are represented by colours ranging from blue (negative correlation, −1), to red (positive correlation, +1). Undefined operational taxonomic units were expressed as the best-hit species with identity (%). The data for the species reads represented 90.4% of the total reads.

**
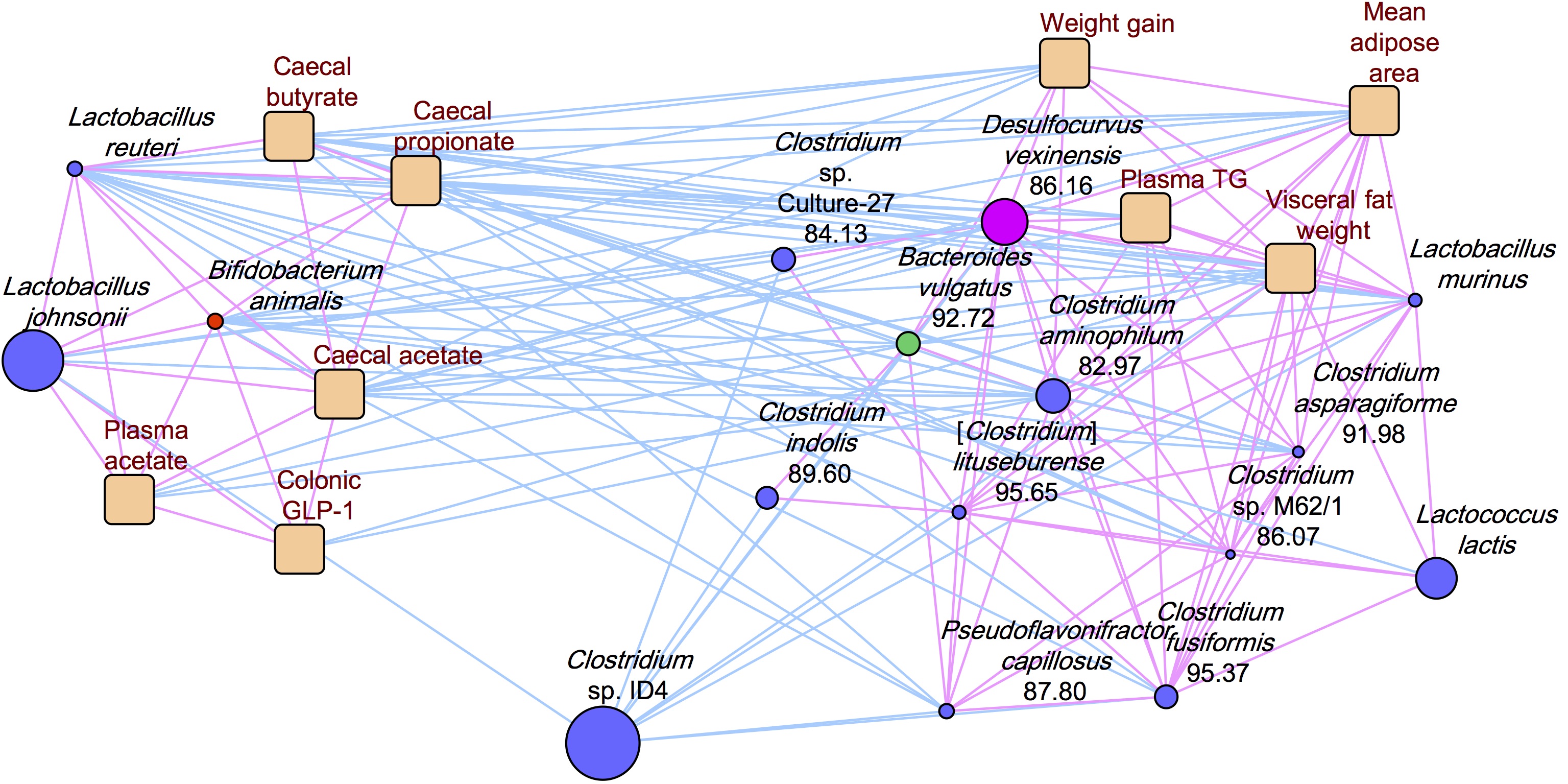
**

**Supplementary Figure S6. Correlation network of predominant operational taxonomic units** (**OTUs), metabolic parameters, and gut metabolites in HFD-fed mice.** Connecting lines in red indicate a positive correlation (Pearson’s correlation coefficient > 0.3), whereas those in blue indicate a negative correlation (correlation coefficient < 0.3). The thickness of the line indicates the extent of the correlation. Nodes in the circle represent the bacterial genera, and the squares reflect metabolic parameters. Undefined OTUs were expressed as the best-hit species with identity (%). OTUs in blue, pink, green, and red circles are Firmicutes, Proteobacteria, Bacteroidetes, and Actinobacteria, respectively. The size of the circle represents the relative abundance of OTUs. The network was visualised using Cytoscape 3.2.1.

**Supplementary Figure S7.** **Experimental design of each animal treatment.** (a) Experiment 1 comprised three groups of C57BL/6J mice fed either a normal diet (D12450B), or HFD (D12451)-diets with or without BlaG treatment. (b) Experiment 2 involved three groups of HFD-fed C57BL/6J mice treated with saline, BlaG, or BloJ. (c) Experiment 3 included two groups of *ob/ob* mice fed a standard diet (CL-2) with or without BlaG.

**Supplementary Table 1.** Primers and programs for analyses of microbiota via quantitative real-time polymerase chain reaction

| Target | Bacterial strain for standard curve | Primer sequence | Program  annealing → extension | References  (Supplemental) |
| --- | --- | --- | --- | --- |
| *Bifidobacterium* | *Bifidobacterium longum*  JCM1217^T^ | F: TCGCGTCYGGTGTGAAAG  R: CCACATCCAGCRTCCAC | 60°C, 20 s → 72°C, 50 s | (1) |
| *B. animalis* ssp. *lactis* | *Bifidobacterium animalis* ssp. *lactis* GCL2505 | F: CCCTTTCCACGGGTCCC  R: AAGGGAAACCGTGTCTCCAC | 60°C, 30 s | (2) |
| *B. longum* ssp. *longum* | *Bifidobacterium longum* JCM1217^T^ | F: TTCCAGTTGATCGCATGGTC  R: GGGAAGCCGTATCTCTACGA | 63°C, 30 s → 72°C, 50 s | (3) |

**Supplementary Table 2.** Primers and programs for expression analyses of mouse genes via quantitative real-time polymerase chain reaction

| Target mouse gene | Primer sequence | Program  annealing and extension |
| --- | --- | --- |
| *Tjp1* | F: GACCAATAGCTGATGTTGCCAGAG  R: TATGAAGGCGAATGATGCCAGA | 60°C, 30 s |
| *Ocln* | F: GGCAAGCGATCATACCCAGAG  R: AGGCTGCCTGAAGTCATCCAC | 60°C, 30 s |
| *Hprt1* | F: TTGTTGTTGGATATGCCCTTGACTA  R: AGGCAGATGGCCACAGGACTA | 60°C, 30 s |

**Supplementary methods**

**Animal treatments**

**Experiment 3**

Five-week-old male C57BL/6JHamSlc-*ob/ob* mice (*ob/ob*; SLC Inc.) were housed in a controlled environment (12-h/12-h light-dark cycle). Mice were fed a non-purified standard diet (CL-2, Clea Inc.), and randomly divided into Ob (n = 10) and Ob+BlaG (n = 10) groups. Mice in Ob+BlaG-treated groups received oral administration of the appropriate *Bifidobacterium* strain (1 × 10^9^ colony forming unit/day) daily for 7 weeks. Mice in the Ob group were given saline daily. OGTT were performed after 6 weeks of administration. After a further week, all mice were sacrificed following a 4 h-fast and blood samples were collected by cardiac puncture. The experimental design is shown in Supplementary Fig. S7c.

**Intestinal permeability test in vivo**

This measure is based on the intestinal permeability of 4000 Da fluorescent dextran–FITC (FD-4; Sigma-Aldrich), as described previously^4^. Briefly, mice were fasted for 4 h before being given FD-4 by gavage (400 mg/kg body weight). After 0, 1, and 2 h, 100 μL of blood was collected from the tip of the tail vein. The blood was centrifuged at at 3,000 × *g* for 15 min at 4°C. Plasma was diluted in a 3-fold volume of PBS (pH 7.4) and the concentration of FD-4 was analysed with a fluorescence spectrophotometer (GloMax; Promega) at an excitation wavelength of 490 nm and emission wavelength of 540 nm. Standard curves were obtained by diluting FD-4 in non-treated plasma diluted with PBS (1:3 v/v).

**Analysis of mRNA expression of intestinal integrity-related genes**

Total RNA was immediately extracted from frozen colon segments via the QuickGene RNA tissue Kit SII (RT-s2; KURABO). Complementary DNA was transcribed using RNA templates with PrimeScript RT reagent Kit (TaKaRa). cDNA samples of intestinal integrity-related genes (*Tjp1*, Tight junction protein-1; *Ocln*, Occludin) were amplified by the Applied Biosystems 7500 Fast Real-Time PCR System (Thermo Fisher Scientific) with SYBR *Premix Ex Taq* II (TaKaRa) using the primers and programs listed in Supplementary Table 2. Expression was quantified in duplicate.

**Supplemental References**

1. Rinttilä T., Kassinen A., Malinen E., Krogius L., & Palva A. Development of an extensive set of 16S rDNA-targeted primers for quantification of pathogenic and indigenous bacteria in faecal samples by real-time PCR. *J. Appl. Microbiol.* **97**, 1166–1177 (2004).
2. Matsuki T. *et al.* Quantitative PCR with 16S rRNA-gene-targeted species-specific primers for analysis of human intestinal bifidobacteria. *Appl. Environ. Microbiol.* **70**, 167–173 (2004).
3. Malinen E., Kassinen A., Rinttila T., & Palva A. Comparison of real-time PCR with SYBR Green I or 5′-nuclease assays and dot-blot hybridization with rDNA-targeted oligonucleotide probes in quantification of selected faecal bacteria. *Microbiology* **149**, 269–277 (2003).
4. Cani, P. D. *et al.* Changes in gut microbiota control inflammation in obese mice through a mechanism involving GLP-2-driven improvement of gut permeability. *Gut* **58**, 1091–1103 (2009).
